# A Case for Hydrogen Sulfide Metabolism as an Oxygen Sensing Mechanism

**DOI:** 10.3390/antiox10111650

**Published:** 2021-10-21

**Authors:** Kenneth R. Olson

**Affiliations:** Department of Physiology, Indiana University School of Medicine—South Bend, South Bend, IN 46617, USA; kolson@nd.edu

**Keywords:** hypoxia, hypoxic vasoconstriction, hypoxic vasodilation, chemoreceptor, carotid body, chromaffin cell, evolution, mitochondria

## Abstract

The ability to detect oxygen availability is a ubiquitous attribute of aerobic organisms. However, the mechanism(s) that transduce oxygen concentration or availability into appropriate physiological responses is less clear and often controversial. This review will make the case for oxygen-dependent metabolism of hydrogen sulfide (H_2_S) and polysulfides, collectively referred to as reactive sulfur species (RSS) as a physiologically relevant O_2_ sensing mechanism. This hypothesis is based on observations that H_2_S and RSS metabolism is inversely correlated with O_2_ tension, exogenous H_2_S elicits physiological responses identical to those produced by hypoxia, factors that affect H_2_S production or catabolism also affect tissue responses to hypoxia, and that RSS efficiently regulate downstream effectors of the hypoxic response in a manner consistent with a decrease in O_2_. H_2_S-mediated O_2_ sensing is then compared to the more generally accepted reactive oxygen species (ROS) mediated O_2_ sensing mechanism and a number of reasons are offered to resolve some of the confusion between the two.

## 1. Introduction: The Need for Oxygen Sensing

A key to survival for aerobic organisms is the ability to detect oxygen availability and make the necessary behavioral, physiological and/or metabolic adjustments to either ensure adequate oxygen delivery or to cope with what is available. These ‘oxygen sensing’ systems operate at various levels, external, internal, tissue-specific, and intracellular. While some form of oxygen sensing system is present in essentially all aerobic (and some anaerobic) organisms, in the interest of space this review is limited to those sensing systems employed by vertebrates to ensure adequate delivery of O_2_ to tissues.

External chemoreceptors monitor ambient oxygen and usually initiate behavioral and physiological responses. Ambient oxygen supply is adequate for most terrestrial organisms but for some it may be limited by high altitude or in poorly ventilated environments such as nests or burrows. Aquatic organisms are more susceptible to ambient hypoxia because the content of oxygen is only 1/30 of that in air, oxygen diffusivity is 200,000 times slower, and the medium is 60 times more viscous. While the percent of oxygen in air is a constant 21%, aquatic organisms can be subjected to wide swings in oxygen seasonally, daily and spatially due to variations in temperature (which affects solubility), photosynthesis (ponds can vary from oxygen saturation to near anoxia in 24 h) and convection (tidal pools, wind mixing, etc. [1]). Additional details on hypoxia in aquatic organisms can be found in Farrell and Brauner [2].

## 2. O_2_ Sensing Systems

Oxygen sensing systems that monitor ambient oxygen would appear to be the first line of defense against hypoxia, but these are relatively rare. The external surfaces of fish gills contain chemoreceptor neuroepithelial cells (NEC; [3,4]) that are anatomically and functionally similar to the neuroepithelial bodies (NEB) near airway bifurcations in lungs of newborn mammals. While gill NEC may continuously monitor O_2_ throughout the life of the fish, NEB appear to be more involved during and shortly after birth in the transition from the relatively hypoxic uterine environment [5]. Surprisingly, there are relatively few other instances of external O_2_ sensors in terrestrial vertebrates as these animals employ internal O_2_ sensors that are better suited to regulate O_2_ stores in the blood and adjust O_2_ delivery commensurate with the needs of the tissues.

Blood-monitoring O_2_-sensing cells are found in all vertebrates. Mammalian neuroepithelial cells and type I glomus cells in the carotid and aortic bodies are essentially identical to NEC cells that line blood vessels in fish gills [4]. This undoubtedly stems from their embryonic origins as the mammalian carotid body and the first gill arch arise from the third embryonic arch and the fourth embryonic aortic arch forms second gill arch and aortic bodies. Mammalian adrenal medullary cells are also homologous to chromaffin cells that line systemic veins in fish. Both cells secrete catecholamines in response to hypoxemia [4,6,7].

Arguably, the most extensively and intensively investigated O_2_-sensing tissues are the blood vessels. It has been generally accepted that hypoxia dilates systemic vessels to match perfusion with metabolism and it constricts pulmonary vessels to match lung perfusion and ventilation. However, this paradigm is not consistent among all vertebrates or even within mammals [8,9,10,11,12]. It may also vary along the length of a single vessel as in the case of the chick ductus arteriosus [13], or with stage of development [14,15], or even over minutes [16]. It was these inconsistencies that led my group to develop the novel hypothesis that the O_2_-dependent metabolism of hydrogen sulfide (H_2_S) was an effective and efficient mechanism to both detect O_2_ availability and to initiate the appropriate downstream effector responses.

## 3. Definition of an Oxygen Sensor

Oxygen is a requirement of all aerobic cells. How cells respond to an oxygen deficiency varies with the cells themselves and with the complexity of their organization into multicellular entities. Cells may respond to a decrease in oxygen availability by altering their utilization of metabolic substrates or decrease their metabolism to conserve resources; mechanisms that are usually more coupled to energy currency than to the actual detection of oxygen per se, e.g., AMP kinase [17].

In the context of this review, an oxygen ‘sensor’ must operate within the bounds of a classical proportional feedback control system in which the ‘sensor’ detects oxygen availability, or tension, and then transduces this information into a signal that can be transmitted by ‘mediators’ (or couplers; [18]) to the appropriate effectors. The strength of the response is proportional to the strength of the stimulus (error signal) and reciprocal mechanisms exist to restore the system as the error signal decreases. Oxygen sensing systems have been described where the ‘sensor’ is an actual oxygen-binding receptor, e.g., prolyl hydroxylases [19,20,21], and the hypoxia-inducible factors (HIFs) serve as mediators. Alternatively, the oxygen ‘sensor’ may be metabolically coupled to oxygen, but not directly (at least not initially) react with it. This is the case for the reactive oxygen species (ROS) theory of oxygen sensing as well as the H_2_S/reactive sulfur species, H_2_S/RSS theory of oxygen sensing. In both of these mechanisms, hypoxia decreases electron flow down the electron transport chain causing either a leak of electrons (ROS theory) or it prevents normal H_2_S catabolism (H_2_S/RSS theory). These ‘sensors’ are unique in that they neither possess an oxygen-binding receptor nor are they involved in general metabolism and they may be more appropriately termed ‘oxygen-coupled sensing systems’.

## 4. Metabolism of H_2_S as an O_2_ Sensing Mechanism

In 2006 we observed that hypoxia and H_2_S produced identical mechanical responses in systemic and respiratory vessels isolated from rats, cows and the most primitive vertebrates, the hagfish and lamprey. We also observed that the hypoxic responses could be inhibited or augmented by inhibitors of H_2_S biosynthesis or H_2_S donors, respectively [22]. Based on these observations we proposed that there was a close inverse metabolic coupling between H_2_S constitutively generated through sulfur metabolism and its O_2_-dependent catabolism, i.e., during hypoxia H_2_S oxidation can no longer keep pace with H_2_S production and H_2_S levels rise. This inexorably couples H_2_S to O_2_ availability. Since then, there has been considerable progress by our laboratory as well as others in developing this hypothesis and in elucidating the many downstream effectors involved in promulgating this O_2_-sensing mechanism. These are described in detail in the following sections.

### 4.1. H_2_S Production and O_2_-Dependent Catabolism

**Figure 1:**
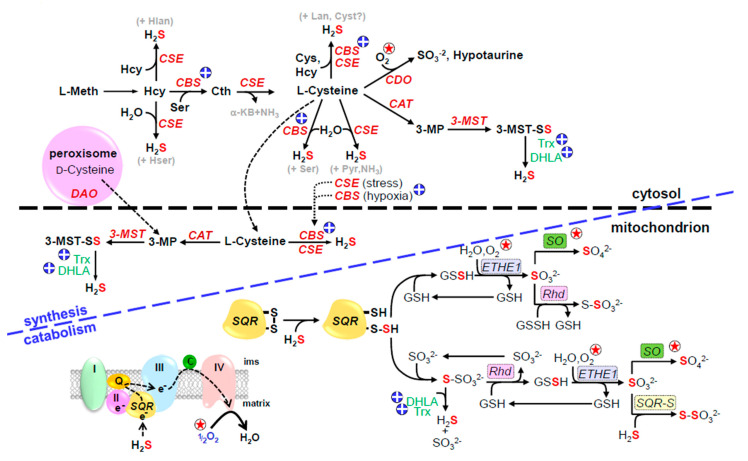
Canonical pathways for H_2_S production and degradation; circled star indicates O_2_-sensitive reactions, circled plus indicates reactions affected by hypoxia-induced increased reducing environment. Cytosolic enzymes, cystathionine β-synthase (CBS) and cystathionine γ-lyase generate H_2_S from homocysteine (Hcy) or cysteine. In addition, cysteine aminotransferase (CAT, also known as aspartate aminotransferase, AST, or glutamate transaminase, GOT) transfers sulfur from cysteine to α-ketoglutarate to form 3-mercaptopyruvate (3-MP) and the sulfur is then transferred to 3-mercaptopyruvate sulfur transferase (3-MST) to form a 3-MST persulfide (3-MST-SS). Both CAT and 3-MST are found in the cytosol and mitochondrion. H_2_S can be liberated from 3-MST-SS by intracellular reductants, dihydrolipoic acid (DHLA), or reduced thioredoxin (Trx). 3-mercaptopyruvate can also be produced from d-cysteine in the peroxisome by d-amino acid oxidase (DAO) which is shuttled to the mitochondrion. During hypoxia CBS migrates from the cytosol to the mitochondrial matrix and in the absence of Lon protease degradation this will generate H_2_S from abundant cysteine in the matrix. CBS activity is also increased as the cell becomes more reduced. General reaction of H_2_S oxidation in the mitochondrion is shown in lower left of figure, blue letters to right show specific reactions. H_2_S is initially oxidized by sulfide:quinone oxidoreductase (SQR) producing a SQR persulfide (SQR-S) and delivering two electrons to the electron transport chain at complex III (III) via coenzyme Q10 (Q). The persulfide sulfur is then transferred to a mobile sulfide carrier, either glutathione (GSH) or sulfite (SO_3_^2−^) and to form glutathione persulfide (GSSH) or thiosulfate (S_2_O_3_^2−^), respectively. The GSSH persulfide is oxidized to sulfite (SO_3_^2−^) by the mitochondrial sulfur dioxygenase (ETHE1) and the sulfur may be further oxidized to sulfate (SO_4_^2−^) by sulfite oxidase (SO). Rhodanese (Rhd, thiosulfate sulfur transferase) reversibly transfers sulfur between thiosulfate and GSSH. Under reducing conditions, H_2_S may be regenerated from thiosulfate by dihydrolipoic acid (DHLA) or reduced thioredoxin (Trx). Reproduced with permission from [23], copyright 2017 Elsevier.

The canonical pathways for H_2_S production, mainly from cysteine, and its catabolism are shown in Figure 1. Essentially all H_2_S production by these pathways is independent of O_2_, whereas nearly all aspects of H_2_S catabolism are O_2_-dependent.

#### 4.1.1. H_2_S Production from Cysteine and Methionine

Four enzymes, cystathionine β-synthase (CBS), cystathionine γ-lyase (CSE aka CGL), cysteine aminotransferase (CAT) and 3-mercaptopyruvate sulfurtransferase (3-MST), and two substrates, l-cysteine (Cys) and l-homocysteine (Hcys), are generally assumed to be the major contributors to cellular H_2_S production. In the transsulfuration pathway, CBS catalyzes the β-replacement of homocysteine with serine which forms cystathionine and commits sulfur metabolism to this pathway [24]. CSE then catalyzes the α-elimination of cystathionine to form Cys, α-ketobutyrate and ammonia (NH_3)_. Both CBS and CSE can then generate H_2_S from Cys via β-elimination reactions. CBS and CSE are relatively promiscuous and H_2_S can also be produced through a variety of reactions involving various combinations of Cys and Hcys [25,26,27,28]. CAT and 3-MST operate in tandem. CAT transfers the amine group from Cys to α-ketoglutarate producing 3-mercaptopyruvate (3-MP) and the sulfur is then transferred to 3-MST forming a persulfide on the enzyme. This sulfur may be then transferred to Cys or glutathione (GSH) forming Cys or GSH persulfides (Cys-S or GSH-S), or the 3-MST-persulfide may be reduced by endogenous reductants such as thioredoxin (Trx) or dihydrolipoic acid (DHLA), thereby liberating H_2_S [29,30,31]. H_2_S can also be derived from d-cysteine in peroxisomes. Here, d-amino acid oxidase (DAO) oxidizes d-cysteine to 3-MP which is then delivered to the mitochondrion by vesicular transport for further metabolism by 3-MST [32]. However, d-cysteine metabolism appears to be limited to the brain and kidney where it protects them from oxidative stress or re-perfusion injury, respectively [32,33].

CBS is predominantly identified in the brain and CSE in the cardiovascular system, although they may be found in other tissues including plasma [10,34,35]. CBS and CSE are generally considered to be cytosolic enzymes, however, CBS may be translocated to the mitochondrion by hypoxia [36] and CSE by stress [37]. CBS also contains two redox-sensitive vicinal Cys (Cys^272^ and Cys^275^) that oscillate between the disulfide and free thiols; reduction of the disulfide increases CBS activity 2-3-fold and increases H_2_S production in HEK293 cells [38]. The redox potential of these Cys (314 mV) are similar to that of cytosolic glutathione (300-320 mV; [38]). This would appear to provide another mechanism to increase H_2_S generation as the intracellular environment becomes more reduced during hypoxia. CBS contains a heme group that when exposed to carbon monoxide (CO) results in inhibition of the enzyme, whereas both nitric oxide (NO) and O_2_ do not appear to be effective at physiological concentrations [39]. Calcium inhibits CSE as well as cytosolic and mitochondrial CAT and this is independent of calmodulin [30,40].

CAT and 3-MST are found in both the cytosol and mitochondria with 3-MST being especially abundant in the mitochondrial matrix [30,41]. The mitochondrial disposition of these enzymes comports well with the presumed mitochondrial locus of an oxygen sensor and the observation that the Cys concentration in the mitochondrion is three-fold greater than that in the cytosol [37] provides additional support for an effective H_2_S-mediated sensing system. In addition, a recent study found that approximately 20% of complex I of the yeast, *Yarrowia lipolytica*, contained an accessory sulfur transferase unit, ST1, whose amino acid sequence was consistent with 3-MST [42]. This subunit also generated H_2_S from 3-MST and the authors suggest its close association with sulfur:quinone oxidoreductase (SQR), the enzyme catalyzing the initial step in H_2_S catabolism (see below), allows for rapid H_2_S detoxification. This would also provide tight coupling between H_2_S and O_2_ in an oxygen sensing system. However, to the author’s knowledge ST1 has not yet been identified in vertebrates.

#### 4.1.2. H_2_S Production from Thiosulfate and Polysulfides

**Figure 2:**
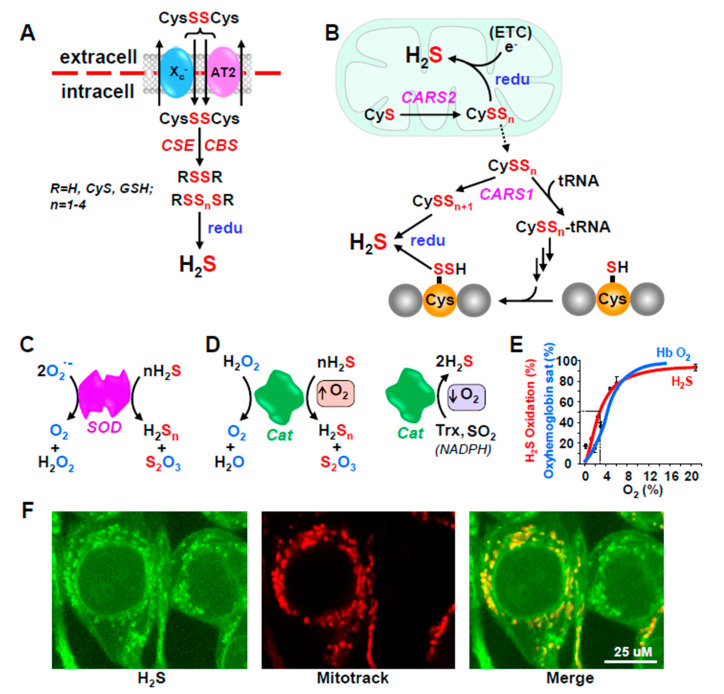
Additional redox-dependent mechanisms for H_2_S production from polysulfides (**A**,**B**) and catabolism by antioxidant enzymes (**C**–**F**). (**A**) Cystine (CysSSCys) is transported into cells by the cystine/glutamate antiporter, system, X_c-_ or sodium-coupled neutral amino acid transporter (AT2). CSE and CBS then catalyze sulfur transfer to produce multiple polysulfides (RSS_n_SR) and H_2_S can be regenerated from these by intracellular reductants (redu) that are expected to increase under hypoxic conditions; the X_c-_ antiporter is also up-regulated by preconditioning with hypoxia. (**B**) Mitochondrial and cytoplasmic cysteinyl-tRNA synthetase (CARS2 and CARS1) catalyze sulfrur transfer from one cysteine to another producing cysteine per- and polysulfides (CysSS_n_; *n* = 1–3). CARS1 also catalyzes attachment of these persulfides to tRNA for subsequent incorporation into nascent proteins. H_2_S can be regenerated from any of these by reductants (redu). (**C**,**D**) Superoxide dismutase (SOD) dismutes superoxide (O_2_^•−^) to peroxide (H_2_O_2_) and water and catalase (Cat) then dismutes peroxide to oxygen and water. Both enzymes oxidize H_2_S to persulfide (H_2_S_2_) and thiosulfate (H_2_S_2_O_3_) in the presence of O_2_. In the absence of O_2_ Cat catalyzes the release of H_2_S from thioredoxin (Trx) or sulfite (SO_3_^2–^) while consuming NADPH. (**E**) The switch of Cat from an oxidase to a reductase is O_2_-dependent with a P_50_ similar to the oxyhemoglobin dissociation curve. (**F**) Mitochondrial origin of H_2_S in HEK293 cells. H_2_S was monitored with the H_2_S-sensitive fluorophore, MeRho-Az and fluorescence co-localized with mitochondria (Reproduced with permission from [48], copyright 2019 John Wiley & Sons Ltd).

There are a number of mechanisms capable of generating H_2_S independent of Cys, Hcy or methionine, where H_2_S is derived from thiosulfate (Figure 1) and polysulfides (Figure 2; reviewed in [23]). Thiosulfate produced as an intermediate in H_2_S oxidation may also release H_2_S when exposed to reductants such as dihydrolipoic acid (DHLA) and this may be catalyzed by thioredoxin (Trx), rhodanese (Rhd), thiosulfate sulfur transferase (TST) or thiosulfate reductase [30,41,43,44,45,46,47]. H_2_S release from thiosulfate has been suggested to serve as an additional source of H_2_S during hypoxia [45].

H_2_S may also be released from persulfides (RSSH; where R may be H, Cys, GSH or a protein thiol) or polysulfides (RSS_n_R; n typically equals 2–5) by DHLA, Trx, GSH and Cys [30,49,50], however, neither NADPH, NADH, GSH, cysteine nor CoA release H_2_S from a 3-MST persulfide [30]. Obviously, these per- and polysulfides could have initially been produced by oxidation of H_2_S, which would serve as a storage or recycling mechanism, but they could also be derived from other sources such as cystine (CysSSCys) or cysteine persulfide (CysSSH; where SH indicates the additional sulfur moiety).

Plasma is relatively more oxidized than cells [51] and most Cys circulates in the bloodstream as oxidized CSSC [52,53]. CSSC is taken up by cells by the cystine/glutamate antiporter, system X_c-_ [54] or by a sodium-coupled neutral amino acid transporter (AT2; [55]; Figure 2A). While conventionally thought of as a source of intracellular Cys, both CSE and CBS catalyze CSSC to multiple polysulfides such as Cys-S_n_H and Cys-S_n_-Cys (where *n* = 1–4); these sulfane sulfur atoms may also be transferred to GSH forming GSH persulfides, e.g., GS-S_n_G ((*n* = 1–3); [55]). These, in turn can be reduced by glutathione reductase (GSR) to generate GS-S_n_H (n-1-3) in μM concentrations [55]. H_2_S can be regenerated from any one of these compounds, especially under reducing conditions. Perhaps as no coincidence, the X_c-_ antiporter is up-regulated in murine stem cells by preconditioning with hypoxia [56], suggestive of an O_2_-sensing process.

Akaike et al. [57], have shown that sulfur can be directly transferred from one Cys to another in a reaction catalyzed by cysteinyl-tRNA synthetase, an enzyme found mainly in the mitochondrion (CARS2) or to a lesser extent in the cytoplasm (CARS1; Figure 2B). This can produce Cys-S_n_H (*n* = 1–3). Cys-S_n_H can be exported from the mitochondria to the cytosol where CARS1 catalyzes its attachment to tRNA resulting in polysulfidation of nascent proteins. This can directly incorporate a redox-sensitive signaling element in a number of regulatory proteins. H_2_S can be released from either the Cys persulfides or persulfidated proteins by cellular reductant processes, as described above. Akaike et al. [57] also propose that electrons leaking from the electron transport chain (ETC) reduce mitochondrial cysteine persulfides thereby liberating H_2_S. This may provide an additional O_2_-sensitive process when O_2_ is low and electrons ‘back up’ in the ETC, although this has not been confirmed.

### 4.2. H_2_S Metabolism (Inactivation)

#### 4.2.1. Conventional Pathways

H_2_S freely diffuses through cell membranes [58,59], and although diffusion out of cells could theoretically contribute to H_2_S inactivation, mitochondrial oxidation is far more efficient, it can be regulated, and it is O_2_-dependent [60]. There is a general consensus that H_2_S is oxidized in the mitochondrion [61]. The initial step in H_2_S oxidation is catalyzed by the flavoprotein, sulfide:quinone oxidoreductase (SQR), whose crystal structure and catalytic activity in humans has recently been identified [62].

SQR is a monomeric integral protein in the internal mitochondrial membrane, facing the matrix, and conveniently situated between complexes II and III of the ETC [62,63]. SQR contains two redox-active cysteines (Cys^201^ and Cys^379^) that are normally present as a disulfide. When H_2_S binds to SQR it is oxidized to sulfane (S^0^) forming a SQR-SH persulfide (SQR-S-SH). Two electrons are transferred via the flavin to ubiquinone (coenzyme Q10; CoQ10) and subsequently delivered to complex III and shuttled down the ETC. The oxygen-dependency of this process is evident; it inexorably links H_2_S catabolism to O_2_ availability and it can serve as a rapid O_2_ sensing system whose response is proportional to the degree of hypoxia.

The sulfane sulfur of SQR persulfide is then transferred to a mobile carrier, either GSH [64] forming GSH-persulfide (GSSH), or sulfite forming thiosulfate [43], the comparatively higher GSH concentrations favoring the former. GSSH is then oxidized by the persulfide dioxygenase, ETHE1, to sulfite (another O_2_-dependent reaction) and further oxidized to sulfate by sulfite oxidase (SO) or another sulfane sulfur from GSSH is transferred to sulfite by rhodanese (thiosulfate sulfur transferase) to form thiosulfate. With sulfite as the mobile carrier, rhodanese transfers the sulfane sulfur to GSH and many of the subsequent reactions are carried out as above. Electrons produced by the oxidation-reduction reaction of water and sulfite, catalyzed by SO, are also delivered to the ETC via cytochrome C, providing yet another pathway that is potentially affected by, and sensitive to, O_2_ availability. Reportedly, human SQR can also form H_2_S_2_ from H_2_S in the absence of a sulfane sulfur carrier [43].

SQR may also catalyze H_2_S oxidation by reverse electron transfer (RET). RET is normally thought to occur when the pool of coenzyme Q (CoQ) becomes over-reduced with electrons from respiratory complex II [65]. These electrons are then transferred retrograde to complex I where some then leak from complex I and reduce oxygen to superoxide which is then dismuted to peroxide thereby signaling elevated ROS and oxidative stress. Electrons from SQR-catalyzed H_2_S oxidation may also be delivered to complex I by RET [66,67]. This has also been proposed to induce superoxide-dependent mitochondrial uncoupling and downstream activation of adenosine monophosphate–activated protein kinase (AMPK; [66]). It also has the potential to increase polysulfide production from H_2_S.

#### 4.2.2. Unconventional Pathways

There are a number of other mechanisms for H_2_S metabolism in addition to the ‘conventional’ pathways described above that could impart O_2_- or redox-sensing attributes. Both the cytoplasmic (Cu-Zn) and mitochondrial (Mn) superoxide dismutases (SOD1 and SOD2, respectively) as well as catalase (Cat) oxidize H_2_S to persulfide (H_2_S_2_) and thiosulfate (H_2_S_2_O_3_) in reactions that require O2 (Figure 2C–E; [68,69]. In addition, in the absence of O_2_, Cat catalyzes the release of H_2_S from thioredoxin (Trx) or sulfite (SO_3_^2−^) while consuming NADPH. This switch of Cat from an oxidase to a reductase is O_2_-dependent with a P_50_ similar to the oxyhemoglobin dissociation curve. Catalase is notably abundant in red blood cells and the O_2_-sensitivity of H_2_S production is suggestive of an O_2_-sensing process designed to couple H_2_S-mediated vasoactivity to O_2_ availability.

We have also shown that numerous polyphenols commonly found in a variety of nutraceutical ‘antioxidants’ (e.g., green tea, berries, grapes and spices) readily oxidize H_2_S to polysulfides and thiosulfate. This activity is the result of O_2_-dependent catalytic redox cycling of the quinone in the B ring of the polyphenol [70,71,72]. Oxidized (ferric) hemoglobin and myoglobin will also oxidize H_2_S to Fe-bound polysulfides and thiosulfate [73,74].

## 5. Inverse and Po_2_-Dependent Relationship between O_2_ and H_2_S

**Figure 3:**
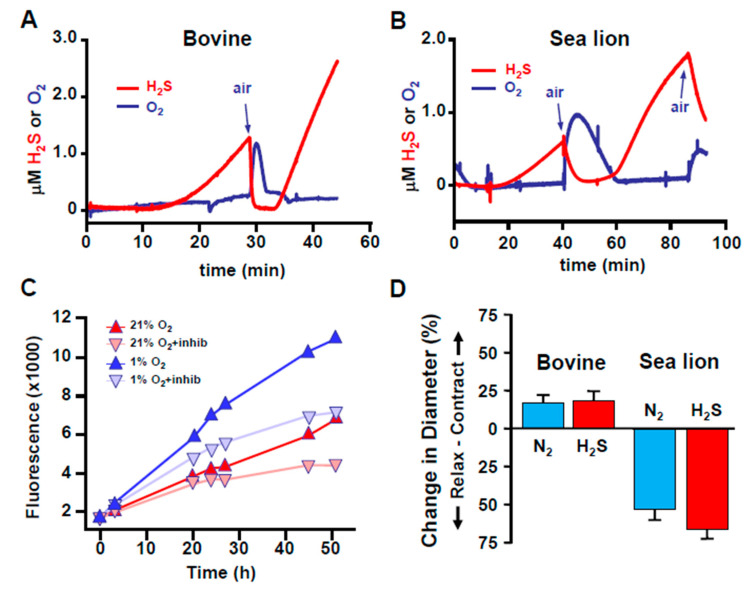
H_2_S production is inversely related to O_2_ acutely and chronically. (**A**,**B**) Relationship between H_2_S and O_2_ in homogenized bovine and sea lion lungs measured in real-time with amperometric electrodes. H_2_S is spontaneously produced in hypoxia, rapidly disappears upon exposure to O_2_, and reappears after the O_2_ has been consumed. (**C**) Comparison of H_2_S production in HEK293 cells in 21% and 5% O_2_ over 52 h monitored with the H_2_S-sensitive fluorophore, AzMC. More H_2_S was produced by cells in 5% O_2_ over this period. Inhibition of H_2_S biosynthesis by cystathionine β-synthase, cystathionine γ-lyase and 3-mercaptopyrucate sulfur transferase with aminooxyacetate, propargyl glycine and compound 3, respectively (+inhibs) decreased but did not prevent H_2_S production in either environment. (**D**) Effects of hypoxia (N_2_) and H_2_S on the diameter of cannulated and pressurized bovine and sea lion pulmonary resistance arterioles. Vessels were slightly precontracted with the thromboxane A2 analog, U-46619 (10^−6^ M) and exposed to either hypoxia (N_2_) or 3 × 10^−4^ M H_2_S. Both N_2_ and H_2_S contracted bovine arterioles but relaxed sea lion vessels. A, B and D from [10]; C from [48], Reproduced with permission from Kenneth R. Olson et al. [10]. Reproduced with permission from [48], copyright 2019 John Wiley & Sons Ltd.

Oxygen and H_2_S do not typically coexist, either in tissues or in the environment. Early studies showed that in a variety of organisms from the mussel, *Geukensia demissa*, to rats that H_2_S is either rapidly consumed in the presence of O_2_ or increases in its absence [75,76,77]. The use of rapid responding H_2_S-selective amperometric electrodes or H_2_S-sensitive fluorophores that provide a long historical perspective of cellular H_2_S production and metabolism has extended these observations to include a variety of tissues and cells from one or more species in all vertebrate classes. It is evident from these studies that the effects of O_2_ on cellular H_2_S can occur within seconds and they may persist for days (Figure 3; [10,48,78,79,80,81,82,83,84]).

**Figure 4:**
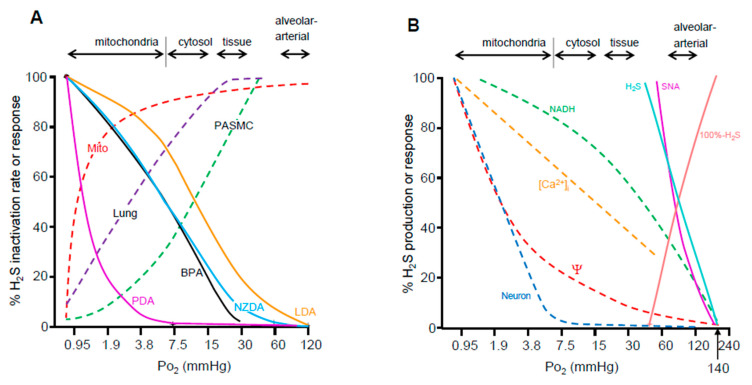
(**A**) Oxygen sensitivity of putative O_2_-sensing systems as a function of approximate range of oxygen tension (Po_2_) in blood, tissues, and intracellular compartments (arrows). These are compared to O_2_ sensitivity of H_2_S oxidation (inactivation) by homogenized bovine lung (Lung), pulmonary arterial smooth muscle cells (PASMC) and bovine heart mitochondria (Mito) indicated by dashed lines. Solid colored lines show physiological responses (hypoxic vasoconstriction) of bovine pulmonary arteries (BPA), lamprey dorsal aorta (LDA), and dorsal aortas from New Zealand and Pacific hagfish (NZDA and PDA, respectively) as a function of Po_2_. The H_2_S oxidation and O_2_ sensitivity of tissue H_2_S consumption is similar to O_2_ sensitivity in vessels from oxygen sensitive vertebrates (bovine, lamprey and New Zealand hagfish), whereas O_2_ sensitivity in Pacific hagfish aortas is considerably lower commensurate with their tolerance to hypoxia. The Po_2_ values at which H_2_S metabolism is impaired are at the low end of cytosolic and mitochondrial Po_2_s and would be expected during hypoxia. It is evident that the efficacy of H_2_S oxidation mechanisms correlates well with physiological responses. (**B**) Comparison of the O_2_ sensitivity of afferent sinus nerve activity from the carotid body (SNA) to H_2_S production (H_2_S; or its calculated inverse, 100%-H_2_S) and components of intracellular signaling in the carotid body (solid lines). The Po_2_ of the half-maximal response (P_50_) for activation of the carotid is essentially identical to the P_50_ for H_2_S production which is more evident when the latter is expressed as the inverse (100%-H_2_S). The P_50_ for intracellular excitation events such as, mitochondrial NADH, intracellular calcium ([Ca^2+^]_I_), mitochondrial transmembrane potential (Ψ) or activation of sympathetic neurons (Neuron; dashed lines) are well below the P_50_s of the intact carotid body or H_2_S production. (**A**) Adapted from [10,88], with permission. (**B**) Adapted from [86], with permission and drawn from data in [87]. Reproduced with permission from Kenneth R. Olson et al. [10]. Reproduced with permission from Kenneth R. Olson et al. [88].

In order for an oxygen-sensing mechanism to be effective it must also be responsive to changes in oxygen tension (Po_2_) experienced by tissues under physiological conditions. Indeed, this appears to be the case as Po_2_-dependent inactivation of H_2_S appears to correlate better with hypoxic vasoconstriction and activation of carotid body chemoreceptors than does the more commonly accepted O_2_-sensing mechanisms (Figure 4). Using amperometric H_2_S sensors we measured the rate of H_2_S oxidation by bovine lung homogenate, bovine pulmonary arterial smooth muscle cells, or purified bovine heart mitochondria as a function of Po_2_ (Figure 4A; [10]). H_2_S oxidation in tissue homogenates begins to fail when Po_2_ falls below 30 mmHg and the Po_2_ at which oxidation is halved (P_50_) occurs in tissues around 4–7 mmHg and in isolated mitochondria below 1 mmHg. These P_50_s are physiologically relevant as they are encountered during hypoxia [85] and they are similar to the P_50_s for hypoxic pulmonary vasoconstriction. It is also noteworthy that the Po_2_ for H_2_S oxidation by isolated mitochondria is strongly left-shifted commensurate with their in-situ environment. The carotid body has a high metabolic rate and a commensurate oxygen sensitivity [86]. O_2_-dependent H_2_S production [87], a corollary of O_2_-dependent H_2_S metabolism, also correlates with O_2_ sensitivity (Figure 4B). Collectively, it is evident from these studies that the reciprocal relationship between O_2_ and H_2_S provides a convenient yin and yang mechanism for oxygen sensing with the caveats that it functions at physiological O_2_ tensions, it responds within seconds, and its effects can be sustained for days.

## 6. Multiple Effectors of H_2_S Metabolism and Signaling Provide a Broad Timeline for O_2_ Sensing

H_2_S and its numerous metabolites arguably comprise one of the most extensive and complex biological signaling systems. This is due in part to the high reactivity of sulfur with itself, as well as oxygen and nitrogen, and in part due to the central and extensive role that receptive cysteines play in a myriad of regulatory proteins that are susceptible to these S/N/O moieties. The chemistry and biology of these signaling process have been the subject of numerous and comprehensive reviews [89,90,91,92,93,94,95,96,97,98,99,100,101,102] and are only briefly summarized below.

### 6.1. H_2_S Signaling via Persulfidation

**Figure 5:**
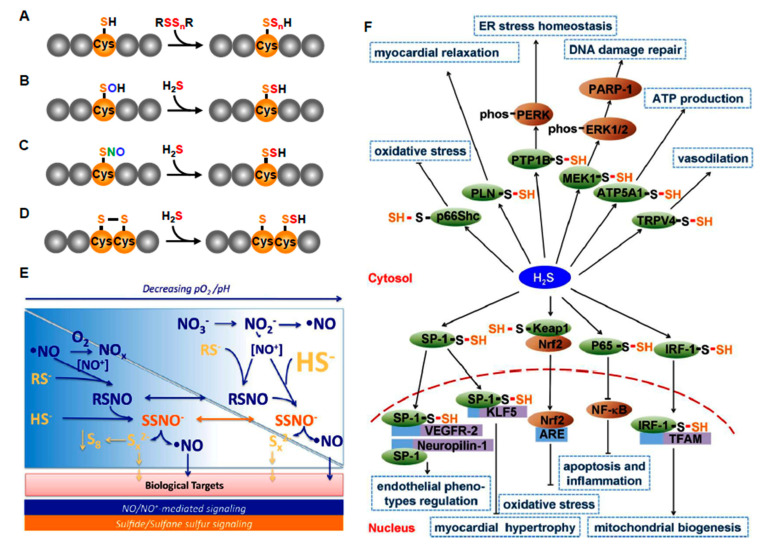
H_2_S and per- polysulfide signaling mechanisms. (**A**–**D**) Interactions of H_2_S or per- polysulfides (RSS_n_R, where R=H, Cys or GSH in various combinations and *n* = 1–4) with reactive protein Cys. (**E**) Sulfur and nitric oxide (NO) signaling. As oxygen falls, cellular pH decreases and H_2_S and NO increase leading to accumulation of a variety of bioactive products. (**F**) Effector pathways for H_2_S (more appropriately per- and polysulfides) signaling in the cardiovascular system. Abbreviations: ATP5A1, ATP synthase subunit α; Cys, cysteine; GSH, glutathione; IRF-1, interferon regulatory factor-1; Keap1, kelch-like ECH-associating protein 1; MEK1, mitogen-activated extracellular signal-regulated kinase 1; NF-κ, nuclear factor κB; Nrf2, nuclear factor E2-related factor 2; PTP1B, protein tyrosine phosphatase 1B; SNO, S-nitrosocysteine; SOH, sulfenyl cysteine; SP-1, specific protein-1; TFAM, mitochondrial transcription factor A; VEGFR, VEGF receptor. (**E**) from [105], with permission. (**F**) From [98], with permission. Reproduced with permission from [98], copyright 2017 The British Pharmacological Society.

Cysteine sulfur is one of the most reactive sulfur-containing small molecules nucleophiles in the cell and as the most highly conserved amino acid, its function in protein (and peptide) structure, catalytic activity and signaling, i.e., the cysteine proteome, is well known [95]. Perhaps the broadest and most extensive mechanism of H_2_S signaling is through persulfidation (also known as S-sulfuration and sulfhydration) of thiols on regulatory protein cysteines. The sulfurs in both H_2_S and protein thiols (Prot-SH) are in their most reduced state and will not react unless one or the other is oxidized. This can occur through a direct reaction between H_2_S and protein cysteine sulfenic acids or protein disulfides, by oxidation of H_2_S to a polysulfide (the oxidized sulfur is referred to as sulfane) that then reacts with a reduced protein cysteine, or by transfer of a sulfane sulfur from a low molecular weight persulfide to the protein thiol (Figure 5A–D). Typically, these reactions inhibit protein function. There are numerous examples of protein persulfidation and it is estimated that at least 30% of cellular proteins are endogenously persulfidated, leaving the opportunity for activation of these proteins by removing the sulfane sulfur with cellular reductants [103]. As more than one sulfur may be attached to these persulfides some protein/peptides may also serve as sulfur reservoirs from which sulfane can be removed and transferred to other proteins by mobile carriers such as cysteine and glutathione. The extent of these processes is an active area of investigation. H_2_O_2_ signaling is essentially similar to persulfide signaling with the caveat that H_2_O is produced when the protein sulfenyl is reduced, whereas the sulfur can be transferred from the protein to another thiol (usually Cys or GSH) and it can be stored or recirculated or reduced to H_2_S which can also be recycled [104].

### 6.2. H_2_S Signaling via Reactions with Nitrogenous Compounds

H_2_S reacts with nitric oxide (NO) to produce a variety of bioactive compounds, some with and some without sulfur including polysulfides, S-nitrosothiols (RSNO, where R = H, Cys, GSH), nitrosopersulfide (SSNO^−^), and nitroxyl (HNO; Figure 5E). Other compounds are likely produced as well, the identity of which is still being actively investigated and debated. NO can also be released from nitrosothiols by H_2_S thereby initiating the NO signaling cascade [106]. Peroxynitrite reaction with H_2_S can form thionitrite (HSNO_2_) and under anaerobic conditions produce HSO and NO or under aerobic conditions, sulfinyl nitrite (HS(O)NO; [107]). H_2_S can react with sulfinyl nitrite to form thiosulfate and nitrite (NO_2_^−^) and the latter may then be reduced by H_2_S, catalyzed by heme iron, and produce NO and nitroxyl (HNO). H_2_S may also directly activate endothelial nitric oxide synthase (eNOS). The production and/or activity of many of these compounds becomes increasingly apparent in hypoxia or as pH falls (Figure 5E), the latter being a common feature of cellular hypoxia [105]. Furthermore, H_2_S and the polysulfide, garlic derivative diallyl trisulfide (DATS) stimulates xanthine oxidoreductase conversion to nitrite reductase with subsequent formation of NO [108]. H_2_S also potentiates the response of soluble guanylyl cyclases to NO and it inhibits phosphodiesterase activity [109].

### 6.3. H_2_S Signaling via Carbon Monoxide

Carbon monoxide (CO) has been shown to inhibit CSE via protein kinase G (PKG)-dependent phosphorylation of Ser^377^ on CSE, thereby inhibiting the production of H_2_S [110]. Hypoxic inhibition of hemeoxygenase-2 (HO-2) can relieve this inhibition and increase H_2_S. In this mechanism H_2_S is proposed to serve as a downstream mediator of the hypoxic response in the carotid body glomus cells, adrenal medulla, and cerebral cortical vessels [111,112,113,114].

### 6.4. Timescale of H_2_S/O_2_ Signaling

As described above, the timescale for H_2_S-mediated stimulus-effector coupling ranges from seconds to days. These responses will depend on factors that are directly involved in affecting cellular H_2_S metabolism as well as those that depend on activating downstream effectors of the hypoxic response. The following sections describe metabolic regulation of H_2_S signaling as well as a few of the downstream effectors with various response rates. In the context of this discussion ‘responders’ refers to those factors that are influenced by hypoxia, ‘regulators’ are those factors that regulate the response and ‘effectors’ are the downstream factors that bring about the desired homeostatic effect.

#### 6.4.1. Rapid Responders

The most rapid H_2_S signaling is expected to be achieved through factors that directly affect H_2_S metabolism and downstream effectors that mediate ion channels.

#### H_2_S Metabolism

(1) Arguably, the fastest of these affect electron transport down the ETC, i.e., decreased availability of the terminal electron acceptor, O_2_ which slows the removal of the constitutively produced H_2_S; our hypothesis of H_2_S-mediated O_2_ sensing [22].

(2) ETHE1, the mitochondrial dioxygenase, ETHE1, catalyzes the oxidization of the mobile persulfide from SQR to form sulfite in a reaction that uses O_2_ and H_2_O. Inhibition of this pathway prevents H_2_S binding to SQR and allows H_2_S and thiosulfate to accumulate [115,116,117].

(3) The other O_2_-dependent reaction, catalyzed by sulfite oxidase (SO), transfers an atom of oxygen from water to sulfite, forming sulfate and reducing SO [118]. The two electrons from SO are delivered to the ETC at cytochrome c thereby inversely coupling sulfite concentration to O_2_ availability. Elevated urinary thiosulfate is common in humans with SO deficiencies [119].

(4) H_2_S can also be ‘regenerated’ from thiosulfate by endogenous reductants in reactions catalyzed by 3-MST or thiosulfate reductase [30,46]. This has been demonstrated in a variety of vertebrate tissues [45]. H_2_S would be expected to increase during hypoxia when thiosulfate accumulates in the mitochondria and the mitochondrial matrix becomes reduced [120]. Thiosulfate also produces K_ATP_ channel-mediated vasodilation which is consistent with the effector of H_2_S vasodilation as discussed below.

#### Rapid Responding Effectors

The variety of effectors of H_2_S signaling is only beginning to be unraveled. Arguably, the best known rapid responding systems are numerous ion channels found in the cardiovascular system and elsewhere. As with most instances of H_2_S signaling, most of the effects are produced by polysulfides. Zhao et al. [121] were the first to demonstrate that H_2_S opened ATP-sensitive potassium (K_ATP_) channels resulting in vasodilation. Subsequent studies have shown that H_2_S also dilates by opening voltage gated Kv7 potassium channels [122]. We initially reported that H_2_S has concentration-dependent multiphasic effects on a variety of blood vessels that are virtually identical to the effects of hypoxia [22]. Furthermore, the degree of hypoxia can further affect H_2_S responsiveness [123]. This effect appears to be explained in part by different effectors, at low concentrations H_2_S constricts systemic vessels by activating Na^+^-K^+^-2Cl^−^ cotransport and opening L-type Ca^2+^ channels, whereas at higher concentrations vasodilation is mediated by K^+^ channels [124]. H_2_S has also been shown to affect a variety of other channels (Figure 5F) including large conductance calcium channels (BK_Ca_) and smooth muscle Ca^2+^ sparks [125,126]. Other effectors include phosphodiesterase inhibition which augments NO responses [127] and decreasing intracellular pH by activation of the Cl^−^/HCO_3_^−^ exchanger [128]. Many of these systems are found in vascular smooth muscle and/or endothelial cells and can result in complex vasoactive effects.

Interactions between H_2_S and NO, described above provide additional examples of rapid activation responses such as activation of TRPA1 channels [129]. These, and other actions of H_2_S in the cardiovascular system have been extensively reviewed [130,131,132,133,134,135].

#### 6.4.2. Medium and Long-Term Responders

Medium to long-term responses include enzymes involved in H_2_S metabolism, effectors that require subsequent catalytic activity. Many also exert action via genomic effectors.

#### H_2_S Metabolism

CBS is transported from the cytosol into the mitochondrial matrix by mitochondrial heat shock protein (mtHsp 70). Under normoxic conditions the prosthetic heme group in CBS is oxygenated which targets it for degradation by Lon protease [36]. Hypoxia prevents this and CBS concentration can double within 10 min and increase sixfold in one hour. CBS is also restored to control levels within 5 min of normoxia. Mitochondrial cysteine concentration is three times that of the cytosol [37]. This CBS-generated H_2_S prevents Ca^2+^-mediated cytochrome C release from mitochondria and prevents mitochondrial swelling while decreasing ROS production. Teng et al. [36] proposed this as a protective effect of H_2_S in myocardial and hepatic ischemia/reperfusion injury.

CSE translocation from the cytosol into the mitochondria is also stimulated by hypoxia in vascular smooth muscle cells. This has been proposed to provide protection from hypoxia by increasing ATP generation from H_2_S [37]. However, this hypothesis is problematic as hypoxia will also decrease electron flux from H_2_S down the ETC, as described above, and vascular smooth muscle can obtain sufficient energy from anaerobic metabolism [136]. Nevertheless, this process could contribute to O_2_ sensing and the resultant hypoxic vasodilation.

Cytosolic cysteine dioxygenase (CDO) uses O_2_ to irreversibly catalyze cysteine oxidation to cysteine sulfinate which prevents cysteine from entering the transsulfuration pathway [137]. Although not directly examined, it is likely that hypoxia will also impair cysteine oxidation and favor H_2_S production. The corollary, CDO deficiency, does, in fact redirect cysteine through this pathway and increases thiosulfate and H_2_S production [138,139], providing some anecdotal support to this hypothesis.

Delivery of substrate may also be affected by hypoxia. Hypoxic preconditioning upregulates the cystine/glutamate antiporter, system X_c-_, in murine neural stem cells, a process that may take from 45 min to 4 h [56].

#### Slow-Responding Effectors

Some of the known reactive thiols on regulatory proteins in the cardiovascular system and their downstream effectors are shown in Figure 5F. Many of them could potentially be affected by per- and polysulfides that result from hypoxia, but this is yet to be thoroughly examined.

## 7. Evidence for H_2_S Mediated O_2_ Sensing in Various Organ Systems and Tissues

*Technical note:* There are numerous reports of H_2_S concentration exceeding 1 μM in body fluids and tissues. These are unphysiologically high [140]. The reader is urged to use caution in interpreting results and conclusions from these studies.

### 7.1. Cardiovascular System

#### 7.1.1. Blood Vessels

Numerous studies on all classes of vertebrates have shown that mono- and multiphasic responses of blood vessels, perfused organs, and intact animals to exogenous H_2_S are similar, if not identical, to those initiated by hypoxia [10,22,81,88,141,142,143,144]. Arguably, the strongest argument for this association, and H_2_S as an oxygen-sensing mechanism, is the comparison of the vasoactive responses of bovine and sea lion pulmonary arteries to hypoxia and H_2_S. Both treatments constrict bovine vessels, but they dilate sea lion vessels, whereas hypoxia increases H_2_S production by both tissues [10].

The effects of compounds that augment or inhibit H_2_S production on vascular responses to hypoxia have had mixed results. Sulfur donors, such as l-cysteine, d-cysteine, 3-mercaptopyruvate and glutathione are reported to augment hypoxic responses of rat aortas [127,145,146], bovine pulmonary arteries [10,22] lamprey aortas [22] and the perfused rat lung [81] and perfused trout gills [143]. Cysteine plus α-ketoglutarate (presumably via the CAT/3-MST pathway) also increases hypoxic vasoconstriction in bovine pulmonary arteries [10,81].

Although inhibitors of H_2_S biosynthesis have inherent problems [147,148], in a number of studies they have been shown to inhibit hypoxic responses in a variety of vessels including the lamprey and rat aorta, bovine pulmonary arteries, perfused trout gills and perfused rat lungs [22,81,143,145,146]. The major pathway for H_2_S production by systemic vessels appears to involve CSE, whereas CBS and CAT/3-MST are involved in H_2_S production in bovine pulmonary vessels; CSE may also be involved in the rat lung [81].

There are a number of studies that suggest additional levels of hypoxic regulation of H_2_S and homeostasis in the vasculature. Intermittent hypoxia associated with sleep apnea decreases CSE and H_2_S in resistance arterioles resulting in increased vascular resistance [149]. Administration of H_2_S to rats with hypoxic pulmonary hypertension (HPH) inhibits the expression of elastin in its extracellular matrix, which also has remarkable regulatory function in forming HPH and remodeling hypoxic pulmonary vascular structure [150]. Furthermore, inhibition of various components of the electron transport chain may also inhibit both hypoxia- and H_2_S-mediated response. The roles of H_2_S in regulation of vascular tone have been recently reviewed [151,152].

#### 7.1.2. Heart

Using a newly developed mitochondria-targeted mass spectrometry probe, Arndt et al. [153] demonstrated that mitochondrial H_2_S was increased in murine hearts after 30 min occlusion of the left anterior descending coronary artery followed by 45 min of reperfusion. Similar results were observed in ischemic liver, supporting the hypothesis that hypoxia increases cellular H_2_S in a variety of tissues and that this has a mitochondrial origin.

CSE has long been presumed to be the primary enzymatic mechanism for H_2_S biosynthesis in the heart [154]. However, recent work suggests important roles for 3-MST. 3-MST is more abundant in cardiomyocytes and smooth muscle than CSE and, curiously, deletion of 3-MST protects young mice from reperfusion injury but exacerbates in in older 3-MST^-/-^ mice and predisposes them to hypertension and cardiac hypertrophy [155]. Additional information can be found in recent reviews [156,157,158].

#### 7.1.3. Central Cardiovascular Regulation

A number of studies have shown that H_2_S modulates the brain cardiovascular centers, and these effects are exacerbated in spontaneously hypertensive animals (e.g., [158,159,160,161]). Several studies have shown an association between H_2_S and brain oxygenation. Sabino et al., [162] reported that microinjection of aminooxyacetate (AOA) into the fourth ventricle of Wis-tar normotensive rats (WNR) did not affect the cardiovascular responses to hypoxia (30 min of 10% inspired O_2_), whereas it blunted the ventilatory and cardiovascular responses in SHR rats. The same group also demonstrated that H_2_S modulates hypoxia-induced hypothermia in rats, and this is also exacerbated in spontaneously hypertensive (SHR) rats due to excess H_2_S production in the caudal nucleus of the solitary tract [163]. These findings support a link between H_2_S and O_2_.

#### 7.1.4. Ischemia/Reperfusion Injury

There is an extensive body of literature on the protective effects of H_2_S and H_2_S donors against ischemia and the efficacy of these compounds to pre- and post-condition the myocardium as well as the central nervous system, liver, kidney and other organs and tissues. While this implies that ischemic conditioning increases cellular H_2_S, which then initiates appropriate effector responses, this is rarely examined and results are on occasion contradictory (e.g., [164,165]). Nevertheless, these studies are indicative of the importance of the O_2_/H_2_S axis in health and disease and are suggestive of the role of H_2_S in O_2_ sensing. The connection between O_2_ and H_2_S is especially important in ischemia and reperfusion injury in the central nervous system, and this is examined in Section 7. Pathophysiological consequences of the H_2_S/O_2_ axis. Additional details can be found in a number of recent reviews [166,167,168,169,170,171,172,173,174,175,176,177].

### 7.2. Respiratory System

#### 7.2.1. General Effects on Respiration

It is well known that high levels of H_2_S inhibit respiration in vertebrates, intravascular injection or inhalation of lower levels of H_2_S will mimic hypoxic hyperventilation in fish, birds and mammals [82,178,179,180,181,182,183,184,185]. These responses appear to be mediated by both central and peripheral mechanisms.

#### 7.2.2. H_2_S and Central Respiratory Centers

H_2_S injected into cerebral ventricles produces a concentration-dependent bradycardia and hypotension, mimicking the diving reflex in mammals [186]. A number of respiratory centers including the pre-Bötzinger (pB) dorsal inspiratory respiratory group, the parafacial respiratory group and hypoglossal rootlets are stimulated by H_2_S [187,188] and H_2_S helps protect the medullary respiratory centers from hypoxic injury [189,190]. 3MST mRNA and protein are expressed in neurons of pre-Bötzinger complex (pre-BotC), hypoglossal nucleus (12N), ambiguous nucleus (Amb) in rats. These 3MST-positive neurons are significantly increased in animals exposed to chronic intermittent hypoxia (CIH) suggesting that adaption to CIH is mediated, at least in part, by H_2_S [191]). Conversely, it has also been reported that microinjection of AOA into the rostral ventrolateral complex (RVLM)/Bötzinger complex increases hypoxia-induced hyperventilation and mitigates hypoxic hyperthermia while injection of H_2_S does not affect ventilation; hypoxia also decreases H_2_S production in rat medullary homogenates [192,193]. Clearly, additional studies are necessary to resolve these issues.

The first pair of gill arches are peripheral chemoreceptors in fish, and they are sensitive to H_2_S (see below). Hypoxic bradycardia is inhibited by removing these arches, but their removal does not affect hypoxic hyperventilation, and inhibitors of H_2_S production, AOA or propargyl glycine (PPG) are also ineffective [82]. This suggests that central chemoreceptors are involved, but it remains to be determined if H_2_S contributes to the O_2_ sensing process in the fish central nervous system.

#### 7.2.3. H_2_S Mediation of Peripheral Chemoreceptors, Carotid Body and Neuroepithelial Cells

Exogenous H_2_S depolarizes carotid glomus cells, it increases afferent nerve activity from the carotid, and it mimics or augments hypoxic hyperventilation while accelerating sinus nerve response to hypoxia [87,194,195,196,197]. Although excess exogenous H_2_S has been reported to inhibit hypoxic responses and to inhibit acetylcholine and ATP release by the carotid body [195,196,198], these are likely to be toxicological, rather than physiological effects.

CBS and CSE immunoreactivity have been identified in glomus cells from cats, rats and mice [87,195,196,198]. In vitro and in vivo studies have provided evidence for CSE, CBS and 3MST-mediation of hypoxia-induced release of H_2_S [87,195,196,197] and hypoxia (Po_2_ ~ 30 mmHg) increases H_2_S production rat carotid bodies [87,196]. Breathing 100% O_2_ will suppress H_2_S-induced hyperventilation suggesting that enhanced O_2_ increases H_2_S metabolism by the glomus cells [185].

Arguably the most extensive studies on H_2_S and O_2_ sensing by the carotid body have been done by Prabhakar’s group (reviewed in; [199,200,201,202]. These authors make the case that H_2_S is a downstream effector of the O_2_ sensing process. They describe two mechanisms, both of which involve relieving tonic inhibition of CSE by carbon monoxide (CO) or nitric oxide (NO) in normoxic conditions. In the primary mechanism, hypoxia inhibits heme oxygenase-2 (HO-2) thereby decreasing carbon dioxide (CO) which relieves CSE inhibition and increases H_2_S synthesis. A backup mechanism has also been proposed where, in the absence of HO-2, neuronal nitric oxide synthase (nNOS) is upregulated and CSE is now inhibited by nitric oxide (NO). As NO synthesis is also O_2_-dependent, H_2_S production will increase as O_2_ falls. The effects of H_2_S on glomus cells have been attributed to inhibition of BK_Ca_ channels [195,203,204], or TASK channels [194], K_ATP_ channels do not seem to be involved [87]. Inhibiting K channels depolarizes the glomus cells, and the resulting influx of calcium initiates release of neurotransmitters. Conversely, it should be noted that other studies have found that chemical inhibition of CSE or genetic deletion of the enzyme had no effect on the hypoxic response [205,206] and clearly additional work is needed.

Peripheral chemoreceptor cells (neuroepithelial cells; NEC), especially prevalent on the first pair of gill arches in many fish, are the antecedents of mammalian carotid glomus cells [4]. NEC contain both CBS and CSE [207]. H_2_S injected into the buccal cavity (mouth) stimulates NEC in the trout gill and produces classic hypoxic bradycardia which is prevented by ablation of the first pair of arches. Both hypoxia and H_2_S depolarize NEC isolated from zebrafish gills, as does hypoxia [82]. NEC are also found on the skin of larval (4-day post hatching) zebrafish, where the gills are relatively undeveloped. Hypoxic responses can be attenuated by chemical inhibition of CBS and CSE in adult zebrafish or by morpholino knockdown of these enzymes in larval forms [207], further implicating H_2_S in O_2_ sensing.

#### 7.2.4. H_2_S Mediation of O_2_ Sensing by Adrenal Medulla

Mammalian adrenal medullary chromaffin cells function as O_2_ sensors during neonatal development [4]. CSE immunoreactivity has been identified neonatal chromaffin cells in both rats and mice and H_2_S appears to mediate hypoxic stimulation of catecholamine secretion in these animals. This likely involves HO-2/CO/CSE and BK_Ca_ channels, similar to those described in the carotid glomus cells [87,111,208].

Fish do not have adrenal glands; however, homologous chromaffin cells line the posterior cardinal vein and anterior kidney, and these cells possess both CBS and CSE; they also release H_2_S and epinephrine into the systemic circulation in response to hypoxia by a CBS-mediated mechanism [209]. Catecholamines are also released by exogenous H_2_S, a process that requires extracellular calcium indicative of chromaffin cell depolarization. It has not been determined if HO-2 and CO are involved in the O_2_ sensing process in these cells.

#### 7.2.5. Airway Receptors

Neuroepithelial bodies in airways sense changes in inspired O_2_ and initiate appropriate cardiorespiratory reflexes [5,210]. These responses are initiated by inhibition of potassium channels, suggestive of a H_2_S-activated response. This is supported by observations that H_2_S enhances airway reflex responses in part, through action on TRPA1 receptors [211]. Low concentrations (0.2%) of inhaled H_2_S also stimulate ventilation in chickens, which has been proposed to be mediated in part by airway receptors [178]. However, a hypoxia-mediated increase in H_2_S in airway receptors has yet to be demonstrated.

#### 7.2.6. Mechanical Effects on Airway Smooth Muscle

Hypoxia and H_2_S relax tracheal and bronchiolar airway smooth muscle, which is mediated, at least in part, by BK_Ca_ channels [212,213,214]. Small airways appear considerably more sensitive to H_2_S than larger ones [215]. Conversely, guinea pig main bronchi and distal trachea are contracted by high concentrations of H_2_S, a response that appears to be mediated by activation of vanilloid neurons [216]. As with airway receptors the direct coupling between hypoxia and an increase in H_2_S remains to be demonstrated.

### 7.3. Kidney

The physiological activities of H_2_S in the kidney have been extensively reviewed [174,217,218]. Low oxygen tensions in the renal medulla have been proposed to necessitate H_2_S responses to help maintain renal blood blow and reduce energy requirements for tubular transport [219] and this is supported by beneficial effects of H_2_S observed in acute kidney injury which occurs during hypoxia or ischemia-reperfusion injury [174]. Additional work on H_2_S/O_2_ coupling is needed to confirm these hypotheses.

### 7.4. Genitourinary Tract

H_2_S has multiple functions in the genitourinary tract [220,221,222]. There are numerous examples where hypoxia and H_2_S relax non-vascular smooth muscle in the genitourinary systems of mammalian and non-mammalian vertebrates [78,223,224,225]. We have recently shown that hypoxia initiates both transient and long-term (days) increases in H_2_S production in HTC116 human colonic epithelial cells [48], suggestive of H_2_S/O_2_ coupled signaling.

### 7.5. H_2_S-HIF Interactions

It is not surprising that H_2_S would interact with hypoxia inducible factors (HIF) in oxygen sensing mechanisms, although the extent and nature of these interactions are still being resolved. H_2_S has been shown to both inhibit HIF-1α expression and stabilization [56,226,227,228,229] and augment it [230,231,232,233,234,235,236]. HIF-1α stabilization also inhibits colonic H_2_S production and may represent a negative feedback mechanism to prevent prolonged HIF-1α stabilization [231]. Recently, polysulfides were shown to inhibit HIF-1α gene expression, protein accumulation and subsequent stabilization with the potency correlated with the number of sulfur molecules, i.e., S_4_ > S_3_ > S_2_; with S_1_ (H_2_S) showing relatively low activity. The main effect of these polysulfides appeared to enhance degradation rather than affect synthesis. The study by Uba et al. [237] is key to our further understanding of H_2_S-HIF regulation as most physiological effects of H_2_S are only initiated after H_2_S is oxidized to polysulfides.

## 8. Pathophysiological Consequences of the H_2_S/O_2_ Axis

Too much of a good thing is not necessarily a good thing and there are a number of instances where hypoxia-initiated increases in H_2_S may be detrimental. The following are three such examples.

### 8.1. Cerebral Ischemia and Stroke

It is well known that the central nervous system is especially sensitive to hypoxic insult. A number of studies have shown that administration of exogenous H_2_S or H_2_S donors or upregulation of endogenous H_2_S production can protect the central nervous system from ischemia and ischemia-reperfusion injury and that the latter is exacerbated by inhibition of endogenous H_2_S production [238,239,240,241,242,243,244,245,246,247]. This would imply that both ischemia and reperfusion injury are associated with a decrease in endogenous H_2_S production. This is supported by several studies that have shown that hypoxia decreases CBS [245,246].

Conversely, other studies have shown that H_2_S worsens the ischemic insult [248,249] and that this is the cause of neuronal death. Although a low Po_2_ has been assumed to be the direct cause of the collapse of the electron transport chain and energy production, Marutani et al. posit that the collapse occurs long before Po_2_ falls to levels that jeopardize O_2_ binding to cytochrome c oxidase (CCO; [249]). Furthermore, they suggested that the relative inability of neurons to metabolize H_2_S may explain this conundrum and, indeed, this seems to be the case. SQR levels in neurons are extremely low, or even non-existent [67,80,250] and CCO is especially sensitive to inhibition by H_2_S with a K_i_ of ~0.2 μM [251]. In a series of elegant experiments Marutani et al. [249] demonstrated in mice, rats, and naturally hypoxia-tolerant ground squirrels that increasing expression of neuronal SQR decreased H_2_S accumulation in the hypoxic brain, sustained energy production and prevented ischemic brain injury. Similar results were observed after administration of exogenous H_2_S scavengers. Conversely, decreasing SQR expression in the brain, heart and liver exacerbated the sensitivity of these tissues to hypoxia. These findings show why the brain is comparatively more sensitive to hypoxia than other tissues and they may offer a therapeutic opportunity in ischemic injury and RPI.

These somewhat contradictory observations may be due to the level and/or the duration of hypoxia or other methodological differences. Clearly, sorting this out and targeting the appropriate H_2_S-metabolizing pathways will have considerable therapeutic value.

### 8.2. High Altitude Pulmonary Edema (HAPE)

High altitude pulmonary edema (HAPE) occurs in un-acclimatized individuals upon rapid ascent to altitudes over 2500 m. HAPE is noncardiogenic and generally attributed to pulmonary vasoconstriction-mediated exudate as a result of sympathetic stimulation, reduced nitric oxide (NO) bioavailability or increased endothelin; inflammatory mediators such as C-reactive protein (CRP) and interleukin (IL-6) may further modulate the disease but do not appear to be the cause [252,253].

Fluid balance across a healthy respiratory epithelium is governed by the rate of salt secretion and reabsorption. Epithelial sodium channels (ENAC) and a basolateral Na^+^, K^+^ ATPase create a alveolar lumen-to-blood trans-epithelial osmotic gradient that reabsorbs fluid which keeps the lung ‘dry’ and ensures a relatively minimal diffusion barrier for respiratory gases. H_2_S inhibits fluid reabsorption by alveolar cells by inhibiting both ENAC and Na^+^, K^+^ ATPase and produces pulmonary edema [254,255,256]. As hypoxia also increases H_2_S production by the respiratory epithelium [254,256], it seems reasonable to assume that this contributes to HAPE. Not surprisingly, pulmonary edema is also a hallmark of H_2_S poisoning in humans (https://www.osha.gov/hydrogen-sulfide/hazards, accessed on date/month/year, accessed on 18 October 2021).

### 8.3. HAPE and Down Syndrome

Down syndrome (DS), the result of trisomy of chromosome 21, results in over-expression of CBS, one of the enzymes encoded on this chromosome [257]. This results in increased H_2_S production in these individuals. In a meta-analysis, Pecze et al. [258] found that there were significantly decreased levels of ATP, CoQ10, homocysteine, serine, arginine and tyrosine; slightly decreased ADP; significantly increased uric acid, succinate, lactate and cysteine; slightly increased phosphate, pyruvate and citrate in DS individuals. They concluded that the levels of metabolites involved in bioenergetic pathways was suggestive of a “pseudohypoxic state” even though arterial gases were normal. With an already elevated titer of H_2_S, one might expect that DS individuals would be especially susceptible to HAPE, at even moderate altitudes, and, indeed, this appears to be the case [259,260].

## 9. Resolving Differences between Competitive Theories of O_2_ Sensing; Reactive Oxygen Species (ROS) vs. Reactive Sulfur Species (RSS)

**Figure 6:**
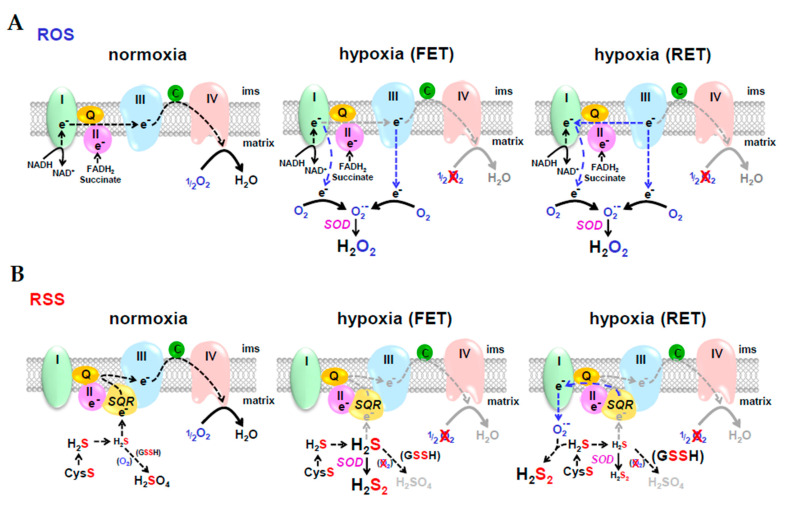
Reactive oxygen species (ROS) and reactive sulfur species (RSS) hypotheses for O_2_ sensing. In the ROS hypothesis (**A**), hypoxia decreases forward electron transport (FET) down the electron transport chain and electrons leak from complexes I and III; in reverse electron transport (RET), electrons are delivered retrograde from complex III to complex I. As electrons leak from the complexes, they reduce O_2_ to superoxide (O_2_^•−^). Dismutation of superoxide, either spontaneously, or catalyzed by superoxide dismutase (SOD), produces hydrogen peroxide (H_2_O_2_) which then diffuses out of the mitochondrion and oxidizes cysteine residues on the appropriate regulatory proteins. In the RSS hypothesis (**B**), hypoxia also decreases FET, but this decreases H_2_S oxidation by sulfide quinone oxidoreductase (SQR) thereby increasing mitochondrial H_2_S concentration. Excess H_2_S is oxidized by SOD, or by electrons from RET, to hydrogen persulfide H_2_S_2_ which diffuses out of the mitochondrion and persulfidates cysteine residues on the appropriate regulatory proteins.

O_2_ sensing by ROS is arguably the most prevalent theory of an O_2_ sensing mechanism, especially in the case of hypoxic pulmonary vasoconstriction (HPV; [261]). In the ROS hypothesis (Figure 6A), hypoxia decreases forward electron transport (FET) down the electron transport chain and as electrons begin to build up they leak from complexes I and III and reduce O_2_ to superoxide (O_2_^•−^). Dismutation of superoxide, either spontaneously, or catalyzed by superoxide dismutase (SOD), produces hydrogen peroxide (H_2_O_2_) which then diffuses out of the mitochondrion and oxidizes cysteine residues on the appropriate regulatory proteins [262]. In reverse electron transport (RET), electrons may be delivered retrograde from complex III to complex I, however, there is little evidence that that this contributes to HPV [263]. In the RSS hypothesis (Figure 6B), hypoxia also decreases FET, but this decreases H_2_S oxidation by (SQR) which then allows mitochondrial H_2_S concentrations to increase. This excess H_2_S is oxidized by SOD, or by electrons from RET, to hydrogen persulfide, H_2_S_2_, which then diffuses out of the mitochondrion and persulfidates cysteine residues on regulatory proteins that are essentially identical to those oxidized by H_2_O_2_.

Many of the arguments in support of ROS in biological signaling can also be made for RSS and it is becoming increasingly difficult to distinguish between the two. Comparisons and distinctions between ROS and RSS have detailed in a recent review [104] and references therein and are only briefly described in the following paragraphs.

### 9.1. Chemical Similarities between ROS and RSS

Both oxygen and sulfur have six valence electrons, but sulfur’s electrons are farther from the positive nucleus which favors electron transfer reactions. Single-electron reduction of O_2_ produces superoxide (O_2_^•−^), hydrogen peroxide (H_2_O_2_), the hydroxyl radical (HO^•^) and water (Equation (1)), whereas single electron oxidation of H_2_S produces a thiyl radical (HS^•^), hydrogen persulfide (H_2_S_2_) and a persulfide “supersulfide” radical (S_2_^•−^) before terminating in elemental sulfur (S_2_; Equation (2)), the latter often cyclizes to S_8_.

H_2_O < ^+e^−^^ HO^•^ < ^+e^−^^ H_2_O_2_ < ^+e^−^^ O_2_^•−^ < ^+e^−^^ O_2_ (1)

H_2_S ^−e^−^^ > HS^•^ ^−e^−^^ > H_2_S_2_ ^−e^−^^ > S_2_^•−^ ^−e^−^^ > S_2_ (2)

Most ROS and much, but certainly not all, RSS signaling is mediated by H_2_O_2_ and H_2_S_2_, respectively; the advantages of the latter over the former were described in Section 6.1 H_2_S signaling via persulfidation.

### 9.2. ROS or RSS?

In addition to their redox similarities there are a number of other disconcerting aspects that hamper evaluation of the relative contributions of ROS and RSS in biological signaling. (1) It can be difficult to analytically distinguish between the two. The redox-sensitive green fluorescent protein (roGFP), arguably, the gold standard for intracellular ROS measurement, is up to 200-fold more sensitive to RSS than ROS; H_2_O_2_ amperometric electrodes are 25 times more sensitive to RSS as well. Other fluorescent ROS probes may also respond to RSS. (2) Most biochemical and physiological experiments are conducted in room air (21% O_2_, or 18.5% O_2_ for cell culture). These conditions well above physiological O_2_ tensions in cells (physioxia) and greatly exceed those in the mitochondrion. This undoubtedly favors ROS over RSS. (3) Life originated in an anoxic and sulfidic world and much of subsequent evolution occurred in these conditions. Many homeostatic pathways were developed under these conditions and likely will become more evident as more experimentation is performed under more physioxic conditions. The reader is referred to the excellent monograph Martin et al. [264] on the evolution of anaerobic energy metabolism in eukaryotic mitochondria.

## 10. Conclusions

H_2_S and polysufide signaling is a relatively new, yet rapidly expanding field. Because of the lability of these sulfur moieties in oxic environments, many challenges remain in identifying relevant signaling species and their metabolism under physioxic conditions. Nevertheless, it is this nearly mutually exclusive relationship between H_2_S and O_2_ that forms the basis for an O_2_ sensing mechanism that is exquisitely tuned to respond to O_2_ availability without the need for complex, highly evolved, and sophisticated sensors. This bespeaks of a mechanism that likely appeared early in evolution and one that has persisted up to the present because of its simplicity and utility.

## Abbreviations

AMP—adenosine monophosphate; AOA—Aminooxyacetate; BKCa—large-conductance Ca^2+^-dependent K^+^ channels; BPA—bovine pulmonary arteries; CBS—cystathionine β-synthase; CCO—cytochrome c oxidase; CIH—chronic intermittent hypoxia; CO—carbon monoxide; CoA—coenzyme A; CSE—cystathionine λ-lyase; DAO—d-amino acid oxidase; DHLA—dihydrolipoic acid; eNOS—endothelial nitric oxide synthase; ETHE1—mitochondrial sulfur dioxygenase; GSH—reduced glutathione; H_2_S—hydrogen sulfide; HIF—hypoxia inducible factor; HNO—nitroxyl; HO-2—hemeoxygenase-2; HS^•^—thiyl radical; HSNO_2_—thionitrite; HS(O)NO—sulfinyl nitrite; Katp—ATP-dependent potassium channels; K_Ca_—Ca^2+^-dependent K^+^ channels; Kv—voltage-gated potassium channels; LDA—lamprey dorsal aorta; MEK-ERK—mitogen-activated protein kinase kinase-extracellular signal-related kinase; Mito—mitochondria; 3-MST—3-mercaptopyruvate sulfurtransferase; mtHsp 70—mitochondrial heat shock protein; NADPH—nicotinamide adenine dinucleotide phosphate; NEB—neuroepithelial body; NEC—neuroepithelial cell; NO_2_^−^—nitrite; NOS—nitric oxide synthase; NZDA—New Zealand hagfish dorsal aorta; PASMC—pulmonary arterial smooth muscle cells; pB—pre-Bötzinger dorsal inspiratory respiratory group; PDA—Pacific hagfish dorsal aorta; Po_2_—partial pressure of oxygen; PPG—propargyl glycine; ROS—reactive oxygen species; SNA—sinus nerve activity; SO—sulfite oxidase; SQR—sulfide-quinone oxidoreductase; TASK—TWIK-related acid-sensitive potassium channels; TRP—transient receptor potential; Trx—thioredoxin; Ψ—mitochondrial transmembrane potential.
